# The role of insulin resistance and *APOE* genotype on blood–brain barrier integrity in Alzheimer's disease

**DOI:** 10.1002/alz.14556

**Published:** 2025-02-24

**Authors:** Alessandro Padovani, Alice Galli, Elena Bazzoli, Chiara Tolassi, Salvatore Caratozzolo, Bianca Gumina, Alberto Benussi, Ilenia Libri, Tiago Fleming Outeiro, Andrea Pilotto

**Affiliations:** ^1^ Neurology Unit Department of Clinical and Experimental Sciences University of Brescia Brescia Italy; ^2^ Neurology Unit Department of continuity of care and frailty ASST Spedali Civili Hospital Brescia Italy; ^3^ Neurobiorepository and Laboratory of Advanced Biological Markers University of Brescia and ASST Spedali Civili Hospital Brescia Italy; ^4^ Laboratory of Digital Neurology and Biosensors University of Brescia Brescia Italy; ^5^ Brain Health Center University of Brescia Brescia Italy; ^6^ Department of preventive and integrative medicine Nutri Neuro Med Desenzano del Garda Italy; ^7^ University Medical Center Goettingen Department of Experimental Neurodegeneration Center for Biostructural Imaging of Neurodegeneration Goettingen Germany; ^8^ Translational and Clinical Research Institute Faculty of Medical Sciences Newcastle University Newcastle Upon Tyne UK

**Keywords:** *APOE* genotype, blood–brain barrier, insulin resistance, permeability

## Abstract

**INTRODUCTION:**

Growing evidence suggests a connection between insulin resistance and apolipoprotein E (*APOE*) genotype in Alzheimer's disease (AD) pathogenesis, but the mechanisms are unclear. We examined effects of insulin resistance and *APOE* genotype on blood–brain barrier (BBB) integrity in AD.

**METHODS:**

BBB integrity was measured in 196 biologically‐confirmed non‐diabetic patients with AD evaluating CSF/serum albumin ratio, kappa and lambda free light chains (FLCs). Insulin resistance was assessed using triglyceride–glucose index (TyG). The impact of TyG on BBB integrity, and its interaction with *APOE* genotypes, was analyzed using multivariate models.

**RESULTS:**

Sixty‐four percent of patients with AD showed altered TyG, with the 21.8% classified as high TyG. TyG subgroups were associated with BBB abnormalities, with similar AD clinical and biomarkers profile. A significant interaction between TyG and *APOE* ε4/ε4 genotype on BBB permeability was found in multivariate analyses.

**DISCUSSION:**

Insulin resistance is a common feature in non‐diabetic AD and correlates with altered BBB permeability, interacting synergistically with *APOE* genotype.

**Highlights:**

- Insulin resistance and apolipoprotein E (*APOE*) genotype are well‐recognized risk factors for Alzheimer's disease (AD).
- Insulin resistance shows high prevalence in patients with AD.
- Insulin resistance is related to damage in blood–brain barrier (BBB) integrity.
- The association between the triglyceride–glucose (TyG) index and BBB permeability varies in relation to *APOE* genotype; patients with the *APOE* ε4/ε4 displayed higher BBB permeability.

## 1 BACKGROUND

Converging evidence from epidemiology, clinical, and biological studies supports a strong relationship between insulin resistance, diabetes, and Alzheimer's Disease (AD).^1^, ^2^

Insulin resistance is defined as a state of decreased responsiveness of target tissues to insulin.^3^ Several lines of evidence suggest that insulin resistance disrupts insulin‐signaling pathways in the brain, leading to impairment in glucose uptake and utilization by neurons. Insulin resistance in the brain can lead to amyloid accumulation, tau hyperphosphorylation, increased oxidative stress, and increased protein glycation.^4^, ^5^ Moreover, insulin resistance may play a significant role in cognitive dysfunction and in the pathogenesis and progression of AD and other neurodegenerative disorders.^6^, ^7^, ^8^, ^9^, ^10^

Several studies have shown that AD is characterized by an increased blood–brain barrier (BBB) permeability,^11^, ^12^, ^13^, ^14^, ^15^, ^16^, ^17^, ^18^, ^19^ and that compromised integrity of cerebrovascular BBB precedes cognitive decline in AD, indicating its potential causal association.^20^, ^21^, ^22^ In fact, the assessment of BBB permeability in vivo poses a significant challenge due to the protected location and complex structure of the brain. Nonetheless, indirect markers such as the cerebrospinal fluid (CSF)/serum albumin ratio have been employed as proxy for BBB integrity.^23^ Likewise, both kappa and lambda free light chains (FLCs), components of immunoglobulins, are synthesized by plasma cells, and a change in their CSF/serum ratio may also reflect changes in BBB permeability.^24^

Among others, the apolipoprotein E (*APOE*) ε4 isoform is a known promoter of BBB dysfunction.^25^ Recently, we highlighted the role of *APOE* in modulating BBB permeability, namely the CSF/serum albumin ratio and kappa and lambda FLCs.^26^ Moreover, insulin regulates the integrity and permeability of BBB through increasing endothelial cell proliferation and expression of tight junction proteins.^27^ However, although in vitro studies have shed valuable light on the impact of diabetes on BBB permeability, there is a paucity of in vivo evidence supporting a role for insulin resistance.^1^, ^4^, ^28^ According to these data and the large evidence of the role of metabolic syndrome in AD, we hypothesised that insulin resistance might affect BBB integrity synergistically with *APOE* genotype. To explore this hypothesis, we measured insulin resistance by using the triglyceride–glucose (TyG) index, which has been shown to be a reliable clinical surrogate marker of insulin resistance. The TyG index has shown good performance in the estimation of insulin resistance compared with the homeostasis model assessment of insulin resistance (HOMA‐IR) index in individuals with and without diabetes, while it does not require insulin quantification and it is independent of insulin treatment status.^29^, ^30^, ^31^, ^32^, ^33^

In this study, we explored the potential role of insulin resistance, assessed by TyG index, in BBB integrity. Furthermore, we explored the relationship between *APOE* genotype and insulin resistance–related damage with respect to BBB integrity, adjusting for vascular and metabolic covariates in a sample of biologically confirmed AD patients.

## 2 METHODS

### 2.1 Participants

This cohort study involved a consecutive sample of participants recruited from the Neurology Unit and the Center for Brain Health of the Department of Clinical and Experimental Sciences at the University of Brescia, Italy.

During the same visit, all patients underwent an extensive standardized evaluation, following standard procedures, and CSF and blood collection. This assessment encompassed a standardized clinical, cognitive, behavioral, and functional protocol, including the Montreal Cognitive Assessment (MoCA) and the Clinical Dementia Rating (CDR‐SB) Sum of Boxes scores^34^ to stratify severity and monitor progression. The presence of neuropsychiatric symptoms was assessed using Neuropsychiatric Inventory (NPI). Brain magnetic resonance imaging (MRI) scans were performed on all patients using either a 1.5 or 3 Tesla scanner to exclude cortical infarcts/hemorrhage or brain tumors. *APOE* genotype was evaluated as reported previously.^26^ Somatic comorbidities were evaluated using the Cumulative Illness Rating Scale (CIRS).^35^

Vascular risk factors, comorbidities, and medication data were evaluated during the clinical assessment. Diabetes was defined as a fasting glucose greater than or equal to 126 mg/dL or the use of diabetes medications. Lifetime diagnosis of hypertension and dyslipidemia and use of antihypertension or hypolipidemic medications were determined by interview. Body mass index (BMI) was collected for all patients. All patients underwent blood collection for standard screening including blood counts, creatinine, folate, thyroid function, fasting glucose, triglyceride, and *APOE* genotype. TyG Index was calculated according to the following formula: TyGindex=ln[(trygliceride∗glucose)]/2, and a cut‐off of 4.55 was used to classify patients with insulin resistance.^32^

Participants satisfying current clinical criteria for probable AD^36^ with positive CSF markers were included (see the Section 2.2 for CSF and cut‐offs further details). Full written informed consent was obtained from all subjects according to the Declaration of Helsinki. The Brescia Ethics Committee approved the study protocol (NP 1471, DMA, Brescia).

### 2.2 CSF standard analyses

CSF was obtained during routine diagnostic lumbar puncture according to a standardized protocol, in the outpatient clinic, at fasting, from 09:30 to 10:30 h. CSF was collected in sterile polypropylene tubes and gently mixed to avoid gradient effects. Routine chemical measures were determined. The remaining CSF was centrifuged for 3 min at 3000 rpm and aliquots (500 mm^3^) were immediately stored at 193.15 K* or in liquid nitrogen for subsequent analysis. CSF total tau (t‐tau), phosphorylated tau‐181 (p‐tau181), amyloid beta (Aβ1‐42 and Aβ1‐40) concentrations were measured by Lumipulse (Fujirebio) by a single experienced technician who was blinded to diagnosis. The internal cut‐off values for AD diagnosis were Aβ42/ p‐tau181 ratio <1.1.^37^

Kappa and λ FLCs, and albumin concentrations in CSF and serum samples were analyzed using the turbidimetric analyzer SPAplus (The Binding Site Group Ltd, Birmingham, UK) with the serum free light chain immunoassay Freelite (The Binding Site Group Ltd, Birmingham, UK) according to the manufacturer's instructions. Only for a subset of patients, intrathecal synthesis of kappa and λ FLCs was determined as published previously,^38^ by the following formulas considering serum FLC concentrations and blood–CSF barrier function:

$$\mathrm{CSF}/\mathrm{serum}\;\kappa \;\mathrm{FLC}\;\mathrm{index}\;=\frac{\kappa \;\mathrm{FLC}s_{\mathrm{CSF}}/\kappa \;\mathrm{FLC}s_{\mathrm{serum}}}{\mathrm{albumi}n_{\mathrm{CSF}}/\mathrm{albumi}n_{\mathrm{serum}}}$$

$$\mathrm{CSF}/\mathrm{serum}\;\lambda \;\mathrm{FLC}\;\mathrm{index}\;=\frac{\lambda \;\mathrm{FLC}s_{\mathrm{CSF}}/\lambda \;\mathrm{FLC}s_{\mathrm{serum}}}{\mathrm{albumi}n_{\mathrm{CSF}}/\mathrm{albumi}n_{\mathrm{serum}}}$$

The following cut‐offs were used to define the presence of an intrathecal kappa FLC (≥6.39) and λ synthesis (≥5.5)^38^; CSF/serum albumin ratio ≥9 was rated as pathologic and positive for BBB damage.^39^

### 2.3 Statistical analysis

Continuous variables are reported as median (interquartile range [IQR]), and categorical variables are reported as numbers and percentages (n, %). Normality distribution of all variables was tested using the Shapiro–Wilk test. Tertile cut‐off values for the TyG distribution were calculated to obtain specific cut‐off values. Thus patients were stratified in three groups with low, intermediate, or high TyG index. Between‐group differences in clinical features were assessed using the Kruskal–Wallis test and chi‐square test for categorical variables, as appropriate. Due to the non‐normality of data, Box‐Cox power transformations of the BBB integrity markers variables were used to correct for the skewness of the residuals, and the appropriate back‐transformation of model β coefficients was performed. Between‐group differences in CSF variables were assessed using univariate models, adjusting for sex, age, and BMI. The interaction between *APOE* and TyG index on BBB integrity was tested using the analysis of covariance (ANCOVA) two‐factor interaction model, with patients being classified according to the number of *APOE* ε4 alleles (0, 1, or 2). Furthermore, to explore the association between insulin resistance, *APOE* genotype, and clinical variables, patients were categorized according to the CDR, MoCA, and NPI scores. Univariate models were employed to test main effects of clinical symptoms and the interaction effect between them, TyG index, and *APOE* ε4 alleles. The correlation between BMI and TyG index, as well as other CSF measures, was explored using Spearman's correlation.

Statistical significance was set at *p* < 0.05 for all tests. Data analyses were performed using JASP version 0.18.1 and R version 4.3.1.

**RESEARCH IN CONTEXT**

1. **Systematic review**: The authors reviewed the literature using traditional (e.g., PubMed) sources and meeting abstracts and presentations. The role of metabolic syndromes in Alzheimer's disease (AD) has been described in several studies. In addition, blood–brain barrier (BBB) permeability is known to be implicated in AD pathology. These relevant citations are cited appropriately.
2. **Interpretation**: Here, in a large sample of biologically confirmed AD patients, we found a significant association between insulin resistance and BBB integrity, with an interaction effect with *APOE* ε4/ε4 genotype.
3. **Future directions**: Understanding of the dynamics of BBB permeability changes in relation to neurodegenerative disease progression is crucial for new perspectives in diagnostic and therapeutic strategies.

## 3 RESULTS

### 3.1 Participant demographics

The study enrolled 196 biologically confirmed AD subjects (mean age ± SD, 71.4 ± 7.2 years; 76 male [38.8%]) (Figure S1). Mean TyG index was 4.570 ± 0.22 in the whole sample, with 64% of the sample showing an abnormal TyG index (i.e., ≥4.55).

TyG index was independent of patients’ age (Spearman's *ρ* = 0.090, *p* = 0.208) and gender (*t* = −0.139, *p* = 0.890), whereas it was correlated with BMI (*ρ* = 0.313, *p* < 0.001). There was a trend of a correlation between TyG index and *APOE* ε4/ε4 genotype (*t* = −1.445, *p* = 0.075). The only two patients carrying the *APOE* ε2 allele were excluded from further analyses, being not representative of this rare subgroup.

Participants were categorized into three TyG groups based on tertiles of the distribution. Demographic, cardiovascular, and metabolic characteristics of included participants are reported in Table 1. Significant differences between groups were observed for BMI and triglyceride and glucose baseline levels, whereas no cognitive/behavioral differences were detected at baseline (Table S1). Thus, age, sex, and BMI were included as covariates of nuisance for further analyses.

**TABLE 1:** Baseline demographic and clinical characteristics of included participants.

	Normal	Intermediate	High	
	*n* = 62	*n* = 72	*n* = 62	*p*‐value
**Demographics**				
Age	71.68 (65.3–77.0)	72.00 (68.0–75.1)	74.00 (69.0–78.0)	0.150
Sex (F/M)	25/13	47/26	18/13	0.765
CIRS, total score	6.00 (3.0–10.0)	6.00 (3.0–10.0)	6.50 (4.0–10.8)	0.546
**Vascular risk factors**				
Hypertension (%)	34 (55%)	31 (43%)	29 (46%)	0.164
Dyslipidemia (%)	41 (66%)	48 (66%)	24 (39%)	0.424
Statin Intake (%)	19 (30%)	28 (40%)	30 (48%)	0.150
Heart disease	0 (0%)	4 (6%)	2 (3%)	0.298
BMI	22.10 (19.8–24.8)	24.00 (22.0–26.4)	24.90 (22.9–26.6)	<0.001^b^
**Biological variables**				
*APOE* ε3/ε4 (%)	28 (45%)	37 (51%)	29 (47%)	0.736
*APOE* ε4/ε4 (%)	5 (8%)	8 (11%)	6 (10%)	0.563
Triglycerides	64.50 (56.0–74.8)	91.00 (83.0‐–98.0)	140.50 (112.0–165.5)	<0.001^a^, ^b^
Glucose	93.50 (86.3–97.8)	97.00 (90.0–105.0)	105.00 (95.0–114.0)	<0.001^a^, ^b^
Creatinine	0.80 (0.7–0.9)	0.84 (0.8–0.9)	0.885 (0.8–1.1)	0.098
TyG index	4.35 (4.3–4.4)	4.55 (4.5–4.6)	4.76 (4.7–4.9)	<0.001^a^, ^b^

*Note*: Data are reported as median (IQR) or *n* (%).

Abbreviations: *APOE*, apolipoprotein E; BMI, body mass index; CIRS, Comorbidity Index Rating Scale; F, females; M, males; TyG, triglyceride–glucose index.

^a^ Low‐TyG ≠ Intermediate‐TyG.

^b^ Low‐TyG ≠ High‐TyG.

### 3.2 TyG associates with CSF core AD and BBB integrity markers

Fluid biomarker levels were categorized by the three TyG groups (Table 2). Insulin resistance severity was not associated with CSF AD pathological hallmarks.

**TABLE 2:** CSF core AD and BBB markers according to TyG stratification.

	Normal	Intermediate	High	
	*n* = 62	*n* = 72	*n* = 62	*p*‐value
***CSF core biomarkers***				
Total tau	728.00 (468.8–989.3)	575.50 (452.3–858.0)	658.90 (459.8–763.3)	0.121
Phosphorylated tau	113.00 (73.3–135.9)	95.50 (79.8–137.2)	91.50 (70.9–131.8)	0.481
Aβ42	494.80 (414.0–611.0)	504.50 (377.0–590.8)	496.50 (410.5–626.3)	0.965
Aβ40	9536.50 (8206.5–13478.3)	10206.50 (7561.8–12977.8)	9256.00 (7232.8–1259.3)	0.749
Aβ42/Aβ40	0.049 (0.04–0.05)	0.046 (0.04–0.05)	0.047 (0.04–0.06)	0.488
Aβ42/p‐tau	4.98 (3.5–6.8)	4.93 (3.5–6.9)	5.24 (3.6–7.1)	0.372
**BBB integrity markers**				
Cells	1.00 (1.0–2.8)	1.00 (1.0–2.0)	1.00 (1.0–3.0)	0.710
Protein	463.50 (332.8–534.5)	430.50 (348.3–537.8)	462.50 (333.8–561.7)	0.774
Albumin CSF/serum	6.40 (5.0–7.9)	6.90 (4.9–8.1)	7.30 (5.3–9.3)	0.032^a^
Altered albumin CSF/serum	19%	19%	46%	0.017
Kappa FLCs CSF/serum^b^	1.65 (1.4–2.4)	1.55 (1.2–2.2)	1.85 (1.3–2.2)	0.305
Altered kappa FLCs CSF/serum	0%	3%	6%	0.668
Lambda FLCs CSF/serum^b^	2.43 (1.9–3.1)	2.54 (1.9–3.4)	2.76 (2.1–3.5)	0.038^a^
Altered lambda FLCs CSF/serum	0%	0%	10%	0.003

*Note*: Analyses were adjusted for age, sex, BMI, and hypertension. Data are reported as median (IQR).

Abbreviations: Aβ, amyloid beta; BBB, blood–brain barrier; CSF, cerebrospinal fluid; FLCs, free light chains; p‐tau, phosphorylated tau;  TyG, triglyceride–glucose index.

^a^ Normal‐TyG ≠ High‐TyG.

^b^ Data are available for a subset of 145 patients.

Regarding BBB integrity markers, we found a significant relationship between TyG index values and CSF/serum albumin levels (*F* = 4.658, *p* = 0.032). The post hoc analysis revealed that albumin was significantly higher in AD patients with high TyG values compared to the other subgroups (*p* = 0.007).

In a subset of 142 patients with kappa and λ FLCs available, we found a significant association between λ, but not kappa, FLCs and insulin resistance severity measured using TyG index (*F* = 4.607, *p* = 0.038). CSF/serum λ FLC levels were higher in AD patients with high TyG values compared to other subgroups (*p* = 0.025) according to the post hoc analysis. No effect of BMI on albumin CSF/serum ratio or kappa or λ FLCs was found.

**FIGURE 1:**
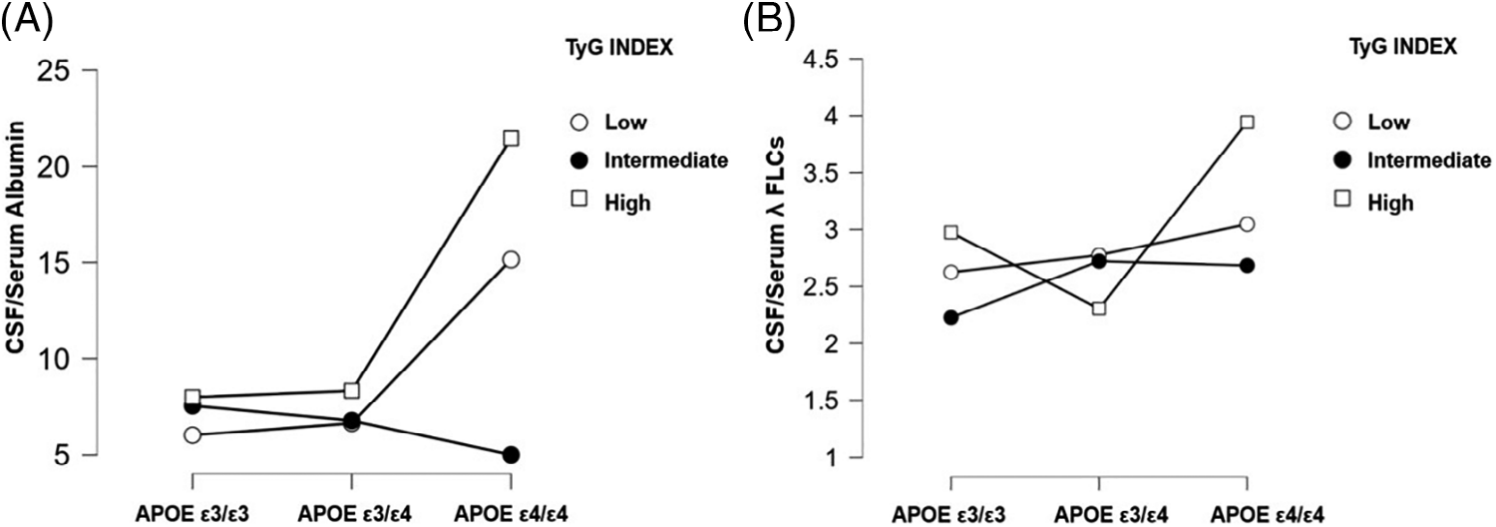
Interaction between *APOE* genotype and insulin resistance on markers of BBB integrity, namely CSF serum/albumin ratio (A) and CSF/serum lambda FLC (B). Analyses were adjusted for age, sex, BMI, and hypertension. *APOE*, apolipoprotein E; BBB, blood–brain barrier; BMI, body mass index; CSF, cerebrospinal fluid; FLC, free light chain.

*APOE* ε4/ε4 genotype was associated with higher CSF/serum albumin levels and CSF/serum λ FLC levels (Table S2). Figure 1 shows the combined effect of *APOE* genotype and insulin resistance on BBB integrity. Specifically, AD patients with *APOE* ε4/ε4 and high TyG index showed significantly higher CSF/serum albumin levels (*F* = 4.753, *p* = 0.001) and CSF/serum λ FLCs levels (*F* = 3.689; *p* = 0.005) as compared to the other AD subgroups.

A significant interaction effect was observed between a more severe cognitive impairment (i.e., MoCA <24) and CSF/serum albumin levels (*F* = 3.464, *p* = 0.034). Moreover, a significant interaction was found between a more severe cognitive impairment and *APOE* ε4/ε4 genotype on CSF/serum λ FLC levels (*F* = 4.168, *p* = 0.004). No significant effect was observed considering disease severity (i.e., CDR) or neuropsychiatric symptoms (i.e., NPI). (See Tables 3–5.)

## 4 DISCUSSION

The present study aimed to investigate the role of insulin resistance measured by TyG index on BBB permeability CSF markers and on AD core–related CSF biomarkers. Furthermore, we explored the interaction of TyG index and *APOE* genotype on either CSF markers of BBB permeability or AD core–related CSF biomarkers. The study was carried out on a consecutive sample of patients with AD and showed that a large proportion of patients displayed pathological TyG index values. Furthermore, our findings revealed a significant correlation between elevated TyG index and increased BBB permeability, exhibiting a clear relationship with *APOE* ε4/ε4 genotype. Notably, although CSF AD biomarkers demonstrated no association with TyG index and *APOE* genotype, there was a strong interaction between *APOE* ε4/ε4 genotype and high TyG index on BBB integrity.

Traditionally, BBB integrity has been assessed in vivo using the CSF/serum albumin ratio, as it is a reliable indicator of BBB permeability because albumin, a relatively large protein (≈67 kDa) synthesized by the liver, does not readily cross the intact BBB.^40^ Under normal physiological conditions, the concentration of albumin in the CSF is much lower than in the serum. However, in instances where the BBB is compromised, the barrier's permeability to substances like albumin increases, leading to a higher CSF concentration relative to serum, thus elevating the CSF/serum albumin ratio. This elevation serves as a clear biomarker of increased BBB permeability and as an indirect, yet effective measure of BBB integrity.

Accordingly, we also observed an increase in the CSF/serum FLCs ratio in individuals with high TyG index, as well as a significant interaction with *APOE* ε4 dosing suggesting that the increased BBB permeability extends to other large molecules, including FLCs. The association with λ but not the kappa FLC is probably due to their different molecular weight, as recently observed also for *APOE* and BBB integrity association.^26^

Previous studies have shown that AD is characterized by an increased BBB permeability, even in the preclinical and prodromal stages of disease.^11^, ^13^, ^14^, ^15^, ^16^, ^17^, ^18^ The relationship between AD and BBB integrity is likely mediated by the *APOE* ε4 allele.^25^ In this regard, it has been reported that the *APOE* ε4 allele might impair BBB integrity through several mechanisms, including the interaction between *APOE* ε4 and low‐density lipoprotein receptor–related protein 1 (LRP1) on pericytes, key cells in maintaining BBB stability. The reduction in LRP1 in endothelial cells, caused by the *APOE* ε4 allele, leads to a loss of important endothelial tight junction proteins, further compromising BBB integrity.^41^, ^42^ In line with these findings, a significant increase in BBB permeability (measured by CSF/serum albumin ratio and kappa and λ FLCs) in relation with *APOE* genotype has been reported.^26^

Moreover, several other factors have been associated with BBB integrity, such as aging,^43^, ^44^ chronic vascular risk factors, type 2 diabetes mellitus (T2DM),^12^, ^45^, ^46^, ^47^ arterial hypertension, dyslipidemia, and hyperhomocysteinemia.^48^, ^49^

Accordingly, a higher BBB permeability was associated with higher levels of glycated hemoglobin and fasting blood glucose levels after adjusting for all confounders in dementia cases.^28^ In fact, a higher BBB permeability was found in individuals with T2DM compared with subjects without T2DM in two different large cohorts.^11^ Furthermore, T2DM was associated with high CSF levels of intercellular adhesion molecule‐1, vascular cellular adhesion molecule‐1, and vascular endothelial‐derived growth factor—CSF biomarkers of angiogenesis and endothelial dysfunction. In animal models, physiological levels of insulin regulate the integrity and permeability of BBB through increasing endothelial cell proliferation and expression of tight junction proteins.^41^, ^42^ Using a murine model of prediabetes, it was shown that the breakdown of BBB integrity precedes the development of cognitive decline and neurodegeneration.^50^ These findings are coherent, with some studies suggesting the pivotal involvement of BBB dysfunction during the onset and early progression of AD.^19^ The mechanisms by which prediabetes compromises BBB integrity and triggers neurodegeneration include heightened inflammation, oxidative stress, pericyte dysfunction, and leukocyte recruitment,^51^, ^52^ possibly affecting amyloid clearence.^53^ Of interest, systemic inflammation induced by LPS injection results in increased permeability of BBB through the loss of tight junction expression and compromised behavior.^54^ In fact, it has been documented that insulin insufficiency and hyperglycemia may alter LDL Receptor Related Protein 1 (LRP1) function, thus decreasing Aβ clearance by modulating tight junction proteins, endothelial cells, and the remodeling of extracellular matrices.^55^ Thus, LRP1‐ induced BBB integrity damage might represent the possible mechanism linking *APOE* genotype and insulin resistance.^56^

Growing evidence supports a role of low chronic inflammation in the pathogenesis of insulin resistance. Clinical conditions of overweight and obesity are in fact characterized by release of free fatty acids and proinflammatory cytokines that eventually might contribute to reduce insulin sensitivity. Of interest, vascular homeostasis is impaired in obesity, a condition in which perivascular adipose tissue (PVAT) releases adipo‐cytokines, leading to oxidation of low‐density lipoprotein and endothelial dysfunction, precisely by promoting disruption of inter‐endothelial junctions, increasing reactive oxygen species and a variety of inflammatory mediators.^57^, ^58^

Thus we postulate that insulin resistance measured by TyG index might contribute to BBB damage in *APOE* ε4 carriers as a result of a low chronic inflammation status, which will need to be tested in larger studies. In this regard, it is of interest to notice that *APOE* produced by microglia seems to be the primary source of *APOE* deposition into Aβ plaques.^59^

In an AD mouse model, *APOE* isoforms in the brain have been reported to impact both Aβ degradation and glucose uptake, particularly by affecting brain insulin/insulin growth factor (IGF) metabolism. Similarly, *APOE* ε4 astrocytes had a poorer glucose metabolism in vitro.^60^, ^61^, ^62^

From a clinical perspective, these findings may have significant implications. First, the variation in BBB permeability, which we found to be related to insulin resistance and *APOE* ε4/ε4, might be relevant for a deeper understanding of the individual response to treatments and side effects of treatment with monoclonal antibodies, known to cause amyloid‐related imaging abnormalities (ARIAs). Second, this study highlights the need to evaluate insulin resistance and prediabetic status in patients with AD, in order to possibly include treatment of this relevant risk factor, particularly in *APOE* ε4 carriers. Homozygous *APOE* ε4 carriers appeared to have an even higher vulnerability, in line with recent reports, highlighting their rarity but unique biological risk for AD.^63^, ^64^ To the best of our knowledge, this is the first study investigating the role of insulin resistance in AD patients by using the TyG index and the first report describing significantly higher TyG among AD patient samples. Previous experimental studies have shown that antidiabetic drugs such as probucol and metformin prevent cognitive deficits by attenuating the neuroinflammation and neurodegeneration mediated through BBB protective properties in a dietary‐induced prediabetic insulin‐resistant mouse model. Thus drugs currently approved to treat metabolic dysfunction hold promise to improve BBB function and reduce the pace of cognitive impairment, as demonstrated in both animal models^65^, ^66^ and clinical trials.^67^ Of note, the glucagon‐like peptide‐1 (GLP‐1) analogue liraglutide crosses the BBB and reduces intrahippocampal amyloid toxicity, significantly increasing memory retention and hippocampal pyramidal neuron numbers in animal models. Moreover, an exploratory trial of the GLP‐1 analog dulaglutide found potential for slowing cognitive decline in patients with T2DM.^68^ Several mechanisms have been advanced to explain the effect of GLP‐1 analogs on dementia, namely the reduction of dementia‐related vascular risk factors^68^ and neuroinflammation.^69^

Finally, the results presented in this study should be interpreted considering certain limitations. First, BBB permeability was inferred using CSF/serum albumin and FLCs ratios which, while informative, are indirect measures. In addition, the study's cross‐sectional nature limits our ability to determine causal relationships or the directionality of the observed associations. Another limitation may be that the present study included only biologically confirmed AD patients, potentially leading to selection bias. Future studies on more diverse neurodegenerative cohorts might be valuable for generalizability of results and to evaluate the potential association between *APOE*, BBB integrity, prediabetes, and diabetes. Moreover, future studies with a longitudinal design might help to understand the temporal dynamics of BBB permeability changes in relation to neurodegenerative disease progression.

In conclusion, our study provides evidence of the role of insulin resistance measured by the TyG in modulating BBB permeability in Alzheimer's disease, in relation to *APOE* genotype, with *APOE* e4/e4 displaying a higher BBB permeability. These findings not only advance our understanding of the pathophysiological mechanisms underlying these diseases but also open new avenues for diagnostic and therapeutic strategies. As the field moves forward, integrating genetic, molecular, and clinical data will be crucial in developing a holistic approach to managing AD. Our study represents a significant step in this direction, offering a new perspective on the interplay between insulin resistance and BBB integrity in the context of AD.

## CONFLICT OF INTEREST STATEMENT

The authors declare no conflicts of interest. Author disclosures are available in the Supporting Information.

## CONSENT STATEMENT

Full written informed consent was obtained from all subjects according to the Declaration of Helsinki. The Brescia Ethics Committee approved the study protocol (NP 1471, DMA, Brescia).

## Supporting information



Supporting Information

Supporting Information
