# Supplementary material for: The role of insulin resistance and APOE genotype on blood–brain barrier integrity in Alzheimer's disease
Source: Alzheimers Dement. 2025 Feb 24;21(2):e14556. doi: 10.1002/alz.14556 (PMC11849409; doi:10.1002/alz.14556)

**SUPPLEMENTARY TABLES**

Supplementary Table 1 – Clinical variables at baseline among the considered groups

|  | Low | Intermediate | High | p-value |
| --- | --- | --- | --- | --- |
| CDR, sum of boxes | 2.83 (3.5) | 2.48 (2.44) | 2.75 (2.39) | 0.796^a^ |
| CDR, global score | 0.75 (0.55) | 0.65 (0.43) | 0.63 (0.34) | 0.315 ^a^ |
| MoCA | 17.82 (6.87) | 17.72 (5.67) | 19.26 (5.48) | 0.570 ^a^ |
| NPI | 10.08 (8.91) | 8.89 (8.73) | 11.89 (11.34) | 0.293 ^a^ |

^a^ P-value were obtained using ANCOVA adjusted for age, sex. Abbreviations: CDR, clinical dementia Rating score; GDS, Geratric depression Scale, MoCA; Montreal Cognitive Assessment; NPI, neuropsychiatric Index.

Supplementary Table 2 – CSF BBB markers according to APOE genotype

|  | APOE ε3/ε3 | APOE ε3/ε4 | APOE ε4/ε4 | p-value |
| --- | --- | --- | --- | --- |
| Albumin CSF/Serum | 6.40 (4.58-9.08) | 6.65 (5.03-7.92) | 7.25 (4.80-8.45) | 0.031^a^ |
| Kappa FLC CSF/Serum | 1.59 (1.32-2.04) | 1.69 (1.21-2.28) | 1.89 (1.29-2.73) | 0.941 |
| Lambda FLC CSF/Serum | 22.67 (5.34) | 22.31 (5.33) | 23.78 (3.99) | 0.045^a^ |

**^a^**= APOE ε4/ε4≠APOE ε3/ε3. Abbreviations: CSF, Cerebrospinal Fluid; FLC, Free Light Chains.

Supplementary Table 3 – Interaction effects between insulin resistance and APOE genotype categorizing patients for disease severity.

| Effect | F | df | p |
| --- | --- | --- | --- |
| ***VD: CSF/Serum Albumin*** | | | |
| CDR | 0.111 | 1 | 0.997 |
| CDR*TyG | 0.231 | 2 | 0.794 |
| CDR**APOE* | 0.432 | 2 | 0.512 |
| CDR*TyG**APOE* | 1.608 | 4 | 0.174 |
| ***VD: CSF/Serum λ FLCs*** | | | |
| CDR | 2.754 | 1 | 0.100 |
| CDR*TyG | 1.656 | 2 | 0.196 |
| CDR**APOE* | 0.067 | 2 | 0.346 |
| CDR*TyG**APOE* | 1.351 | 4 | 0.262 |

Abbreviations: CDR, Clinical Dementia Rating Scale; APOE, Apolipoprotein E; TyG, Tryglicerides glucose index

Supplementary Table 4 – Interaction effects between insulin resistance and APOE genotype categorizing patients for cognitive impairment.

| Effect | F | df | p |
| --- | --- | --- | --- |
| ***VD: CSF/Serum Albumin*** | | | |
| MoCA | 0.817 | 1 | 0.367 |
| MoCA*TyG | 3.464 | 2 | **0.034*** |
| MoCA**APOE* | 0.369 | 2 | 0.691 |
| MoCA*TyG**APOE* | 1.834 | 4 | 0.126 |
| ***VD: CSF/Serum λ FLCs*** | | | |
| MoCA | 2.279 | 1 | 0.134 |
| MoCA*TyG | 1.235 | 2 | 0.295 |
| MoCA**APOE* | 1.614 | 2 | 0.204 |
| MoCA*TyG**APOE* | 4.168 | 4 | **0.004*** |

Abbreviations: MoCA, Montreal Cognitive Assessment; APOE, Apolipoprotein E; TyG, Tryglicerides glucose index

Supplementary Table 5 – Interaction effects between insulin resistance and APOE genotype categorizing patients for neuropsychiatric symptoms.

| Effect | F | df | p |
| --- | --- | --- | --- |
| ***VD: CSF/Serum Albumin*** | | | |
| NPI | 1.186 | 2 | 0.309 |
| NPI*TyG | 0.620 | 2 | 0.649 |
| NPI**APOE* | 3.119 | 2 | **0.017*** |
| NPI*TyG**APOE* | 0.729 | 6 | 0.626 |
| ***VD: CSF/Serum λ FLCs*** | | | |
| NPI | 0.221 | 2 | 0.802 |
| NPI*TyG | 1.130 | 4 | 0.345 |
| NPI**APOE* | 0.443 | 4 | 0.723 |
| NPI*TyG**APOE* | 1.116 | 4 | 0.359 |

Abbreviations: NPI, Neuropsychiatric inventory ; APOE, Apolipoprotein E; TyG, Tryglicerides glucose index

Supplementary Figure 1- Association between MoCA and CSF values


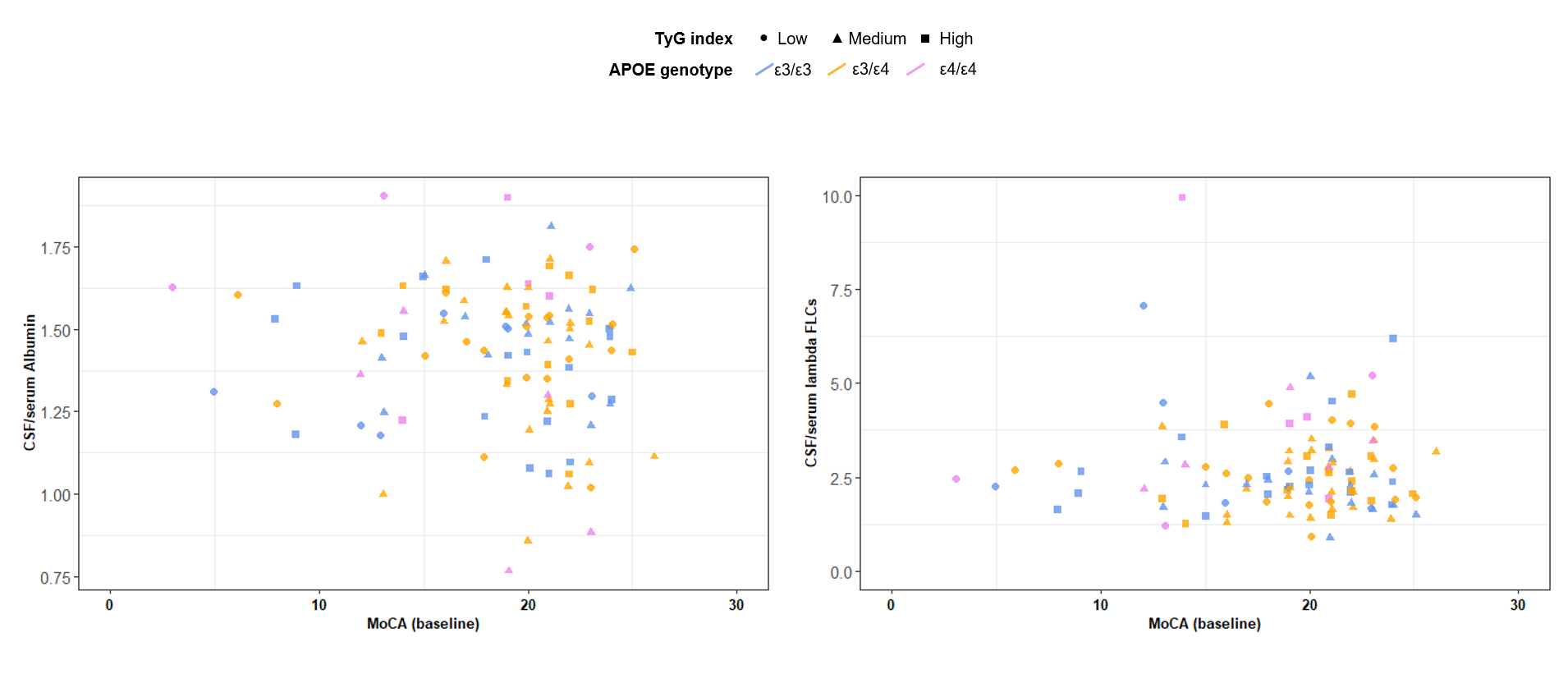

Supplement: Supplementary file 1 — Supporting Information [file ALZ-21-e14556-s002.docx]
